# Analysis of hair cortisol levels in captive chimpanzees: Effect of various methods on cortisol stability and variability

**DOI:** 10.1016/j.mex.2016.01.004

**Published:** 2016-01-16

**Authors:** Yumi Yamanashi, Migaku Teramoto, Naruki Morimura, Satoshi Hirata, Juri Suzuki, Misato Hayashi, Kodzue Kinoshita, Miho Murayama, Gen’ichi Idani

**Affiliations:** a Wildlife Research Center, Kyoto University, Japan; b Primate Research Institute, Kyoto University, Japan; c National Institute for Environmental Studies, Japan

**Keywords:** Hair cortisol, Animal welfare, Chimpanzee, Practical methodology, Long-term stress

## Abstract

Hair cortisol has been reported to be a useful measure of long-term hypothalamic–pituitary–adrenal (HPA) axis activation in several species. It serves as a practical tool for long-term stress assessment, but it is important to understand the methodological factors that can affects hair cortisol assays to avoid methodological artifacts. To that end, we tested several procedures for measuring cortisol levels in hair collected from captive chimpanzees. The results showed that reproducibility was high, and we found no differences in cortisol levels among the various storage, drying, and sampling methods. However, the fineness of homogenized hair, sample weight, and extraction time affected absolute hair cortisol concentration. Although hair cortisol levels were stable over time, factors that may influence measurement results should be kept constant throughout a study.

• We modified and validated a methodology involving enzyme immunoassays to reliably measure the hair cortisol levels of captive chimpanzees.
• The results revealed that the fineness of homogenized hair, sample weight, and extraction time caused variations in absolute hair cortisol concentrations in chimpanzees. In contrast, storage, drying, and sampling from similar body parts did not affect the results.

## Graphical abstract

Summary of the experimental process. The items colored blue affected the results of the hair cortisol analysis in the present study. The stippled items were found to affect the results obtained in our previous study [1].

**Figure:**
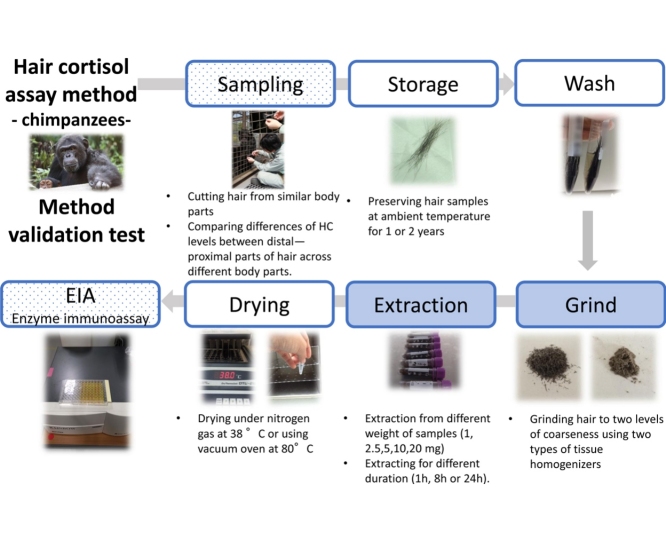

## Method details

Samples were collected from chimpanzees by cutting arm hair (approximately 200 mg) with scissors. Samples were washed with 5 ml isopropanol by shaking the tubes three times for 2 min each. After drying, the samples were stored at ambient temperature until analysis. Then, about 150 mg of the sample was ground into a fine powder for 4 min at 6500 rpm with a Precellys 24 tissue homogenizer (Bertin Technologies, Orléans, France). The powdered samples were weighed (minimum 5 mg) and placed in 2-ml tubes, and 1-ml methanol was added. Cortisol was extracted by shaking the tubes for 24 h at ambient temperature. Following extraction, the samples were centrifuged, and 0.6 ml of supernatant was aliquoted into different tubes and evaporated by vacuum oven at 80 °C. Samples were reconstituted using phosphate buffer, and cortisol concentrations were measured using a commercially available enzyme immunoassay (EIA) kit (Salivary cortisol, Salimetrics LLC, Philadelphia, PA, USA). These methods to quantify hair cortisol levels were based on our previous study, which was modified according to the method-validation results presented below [1].

### Method validation

We investigated the effects of various sampling, grinding, and drying methods and storage and extraction times on the analysis of hair cortisol. In addition, we checked the minimum number of samples necessary to estimate the average hair cortisol level during a year. Samples were collected from 72 captive chimpanzees (38 males and 42 females) living in the Kumamoto Sanctuary (KS), the Primate Research Institute (PRI), and the Great Ape Research Institute. (For information about care and husbandry of these chimpanzees, see Refs. [2], [3], [4]). Following the procedure described in the Methods section, except when we tested the grinding process, we used a Precelly 24 tissue homogenizer for grinding hair. According to the instructions, the minimum concentration of cortisol that can be measured by this kit is 0.07 ng/ml. Intra-assay variability was 5.12%, and the inter-assay variability values for high and low controls were 4.88 and 7.17%, respectively (mean of nine plates). The results from all of the validation tests are shown in Table 1 and Supplementary Material.

#### Statistical analysis

Pearson's correlation was used to test the correlation between samples. Paired *t*-tests or a one-way analysis of variance (ANOVA) was used to assess differences between samples. Tukey's HSD tests were performed as a post hoc test following the ANOVA to examine pairwise differences. All statistical tests were conducted using R 3.1.0. [5]. Data on hair cortisol concentrations were log-transformed prior to statistical analysis.

**Fig. 1:**
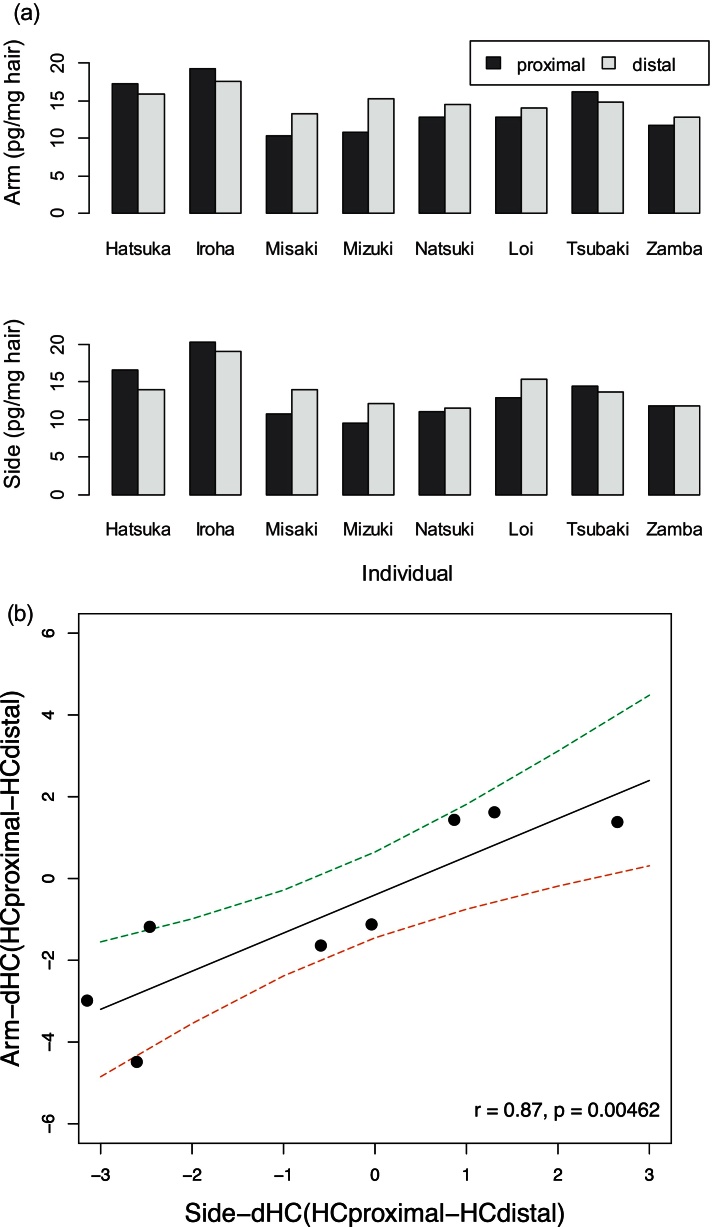
Differences in hair cortisol levels between proximal and distal segments of hair (a: dHC) and correlation between dHC obtained from arm hair and side hair (b). The solid line represents the fitting line, and the dashed lines indicate the 95% confidence interval.

(a) Effects of sampling location
  Our previous study [1] reported variation in hair cortisol levels across different body regions. To reduce this variation, we suggested using a consistent body region for multiple samplings. We further assessed variability in the sampling procedure by collecting hair from similar areas on the chimpanzees’ arms twice during the same day.
  Collecting samples from the arm region twice in one day did not significantly affect hair cortisol concentration (*t* = 0.078, df = 13, *p* = 0.94); moreover, the hormone levels in the two samples were significantly correlated (*r* = 0.70, *p* < 0.01). However, the coefficient of variation (CV (%): 100 × standard deviation of the two samples/mean of the two samples) was higher than that of the other factors. Therefore, although collecting the samples from similar body regions enabled us to monitor hair cortisol levels in the long term, we need to consider the small variations that may result from sampling.
  Furthermore, we checked whether segmental differences were similar across different body regions. We collected hair from an arm and the side of eight chimpanzees. We cut each hair sample into distal and proximal parts and processed them separately. We calculated the difference in hair cortisol concentration between the distal and proximal parts of the hair (dHC) by subtracting the hair cortisol level of the distal part (HCdistal) from that of the proximal part (HCproximal).
  Consistent with our previous study [1], there were variations in the direction of the differences in the cortisol concentrations found in the proximal and distal sections of hair segments. Cortisol concentrations in the distal sections were higher than those in the proximal sections in five of eight samples, whereas the difference was in the reverse direction in the remaining three samples (Fig. 1a). In addition, a significant correlation was detected between individual dHC obtained from arm hair and that obtained from the side of the body (Fig. 1b: *r* = 0.87, *p* < 0.01). These results indicated that we can obtain reliable information by sampling similar body parts, even though absolute hair cortisol concentrations differ across different body regions [1].
(b) Effects of storage time under ambient temperature
  To check whether storage time affected the results, we assessed the same samples twice within 1 month and again after 1 or 2 years; we then compared the results.
  Results indicated high reproducibility (*r* = 0.92, *p* < 0.0001), and no significant differences were found between samples processed at different times (*t* = −1.2, df = 21, *p* = 0.25). Cortisol levels did not differ between hair samples preserved for 1 and those preserved for 2 years at ambient temperature (Pearson's correlation: 1 year, *r* = 0.76, *p* < 0.0001; 2 years, *r* = 0.90, *p* < 0.0001; paired *t*-test: 1 year, *t* = −1.3, df = 28, *p* = 0.19; 2 years, *t* = −0.64, df = 15, *p* = 0.53).
(c) Fineness of homogenized hair
  We tested two types of tissue homogenizer to assess the impact of degree of grinding: a Precellys 24 tissue homogenizer for 4 min at 6500 rpm (Bertin Technologies, Orléans, France), which grinds samples to a fine powder, and a bead beater-type homogenizer (Beads Crusher μT-12; TAITEC Co., Ltd., Saitama, Japan), which produces a coarser powder.
  Hair cortisol levels were 1.5-fold higher in the finely ground than in the coarser samples (*t* = −5.4, df = 18, *p* < 0.0001), although the values for the two homogenizers were significantly correlated (*r* = 0.85, *p* < 0.0001). Grinding should be kept constant to maximize extraction efficiency and reproducibility. Although the increase in homogenizing duration may have increased the fineness in the Beads crusher, it sometimes resulted in the destruction of homogenizing tubes before reaching a similar level of fineness.
(d) Minimum weight requirement of hair samples
  To check the minimum weight requirement of hair samples, we divided samples into 1-, 2.5-, 5-, 10-, and 20-mg groups after grinding and extracted the cortisol. We then compared the results obtained from different groups.
  Hair cortisol levels assessed from different sample weights showed significant variation (ANOVA: *F*(4, 20) = 27.2, *n* = 6, *p* < 0.001). Post hoc tests revealed that results obtained from 1 mg of hair were significantly higher than those obtained from 2.5, 5.0, 10, and 20 mg of hair (Tukey's HSD: *p* < 0.001). Furthermore, results obtained from 2.5 mg of hair were significantly higher than those obtained from 20 mg of hair (Tukey's HSD: *p* < 0.05). The difference between the results obtained from 20 mg and that from 5.0 mg of hair was not significant, but it supported the same trend (*p* = 0.067). In addition, the correlations between results obtained from 1-mg samples and other quantities occasionally did not reach significance (20 mg, *r* = 0.68, *p* = 0.13; 10 mg, *r* = 0.84, *p* = 0.03; 5 mg, *r* = 0.79, *p* = 0.06; 2.5 mg, *r* = 0.80, *p* = 0.05). Other quantities were correlated (*r* = 0.96–0.98, *p* < 0.01). The average cortisol concentration measured in the assay plate was 0.272 ng/ml for 1.0 mg of hair, which was higher than the lower limit of the sensitivity of the kit (0.07 ng/ml). The minimum readability of the balance (A&D HR-200) used to weigh hair was 0.1 mg. Although 1.0 mg was higher than this minimum value, it is possible that small samples enable less precision. Therefore, at least 5 mg (and preferable 10) of hair was needed considering the differences in the absolute values and the correlation results.
(e) Extraction time
  We checked the effects of extraction time on the results. Cortisol was extracted by shaking tubes containing samples and 1.0 ml methanol for 1, 8, or 24 h. We then compared the results.
  Extraction time had a significant effect on hair cortisol levels (ANOVA; *F*(1, 24) = 33.3, *p* < 0.0001). The post hoc test revealed that increased extraction time produced a slight but significant increase in hair cortisol concentrations (24 h vs. 8 h: *p* = 0.035, 8 h vs. 1 h: *p* < 0.01).
(f) Effects of drying method
  We compared two drying methods: drying under nitrogen gas at 38 °C (Dry thermo unit, TAITEC, Saitama, Japan), used in the previous report [1], [6], and in a vacuum oven at 80 °C for 2 h (Yamato Scientific Co., Ltd., Yamanashi, Japan).
  The drying methods tested (nitrogen gas at 38 °C vs. vacuum oven at 80 °C) did not produce significantly different results (Pearson's correlation: *r* = 0.76, *p* < 0.0001; paired *t*-test: *t* = 1.2, df = 25, *p* = 0.23).
(g) Intra- and inter-individual variability
  We analyzed hair from 72 chimpanzees in KS and PRI three to four times per year to determine intra- and inter-individual variability and to establish the minimum number of samples necessary to estimate the average hair cortisol level during a year.
  The mean intra- (30.1%) and inter- (24%) individual variations in hair cortisol levels were higher than differences among the methodological procedures. Importantly, this means that our method overcame the technical fluctuation and yielded results showing that differences in hair cortisol levels can reflect the endocrinological status of animals.
  The number of samples collected during the year had a significant effect. The hair cortisol levels in individual chimpanzees fluctuate widely throughout the year; thus, the correlation among samples collected at various times during the year varied (correlation coefficient range, 0.26–0.70). We found that averaging two values produced similar results to averaging four values. Thus, the mean value of two or three hair samples was highly correlated with that of four samples (one vs. four samples: *r* = 0.55–0.88; two vs. four samples: *r* = 0.88–0.94; three vs. four samples: *r* = 0.97–0.99; all *p* < 0.05, two-tailed). Therefore, two samples are sufficient for estimating the average hair cortisol level throughout a year. In the future, we will use this methodology to investigate the factors influencing hair cortisol level to improve the quality of captive care of these animals.

#### Results summary

Hair cortisol levels were stable across several measurement protocols, suggesting that the assay is a reliable and practical tool for monitoring long-term stress. The minimum weight requirement for hair samples was 5 mg (preferably 10), which corresponds to 5–7 hairs (10–15 hairs for 10 mg), although cutting more hair at the time of sampling and using parts of it for extraction can produce stable results given the variation according to body region. Intra- and inter-individual hormone variations were higher than variations across methods. However, the present findings together with those of our previous study [1] revealed that the degree of grinding, extraction time, body part, hair color, and various EIA systems caused variations in absolute hair cortisol concentrations in chimpanzees. These variables should be kept constant and considered when planning and interpreting the results of hair hormone analyses. In contrast, storage, drying, and sampling from similar body parts did not affect absolute hair cortisol levels. Differences in hair cortisol levels between proximal and distal segments showed a consistent trend across different body regions. Thus, hair cortisol analysis can be performed in various laboratory settings and with hair obtained from various animal care facilities providing the causes of variations are taken into consideration. The results are promising in terms of understanding the relationship between long-term stress and stress-related issues in captive animals.

## Additional information

### Background

Long-term stress may negatively affect animal welfare [7]. Several examples of abnormal behavior, reproductive failure, and certain types of illness (e.g., cardiovascular diseases) reported in captive animals may be related to stress in the captive environment [8], [9], [24]. Given the longevity and long developmental period of chimpanzees [3], [10], it is important to monitor their stress level over the long term. However, the practical limitations of the methods used have prevented the estimation of long-term stress levels in captive wild animals.

Hair cortisol has been shown to be a useful measure of long-term hypothalamic–pituitary–adrenal (HPA) axis activation in several species [6], [11], [12], [13], [14], [15], [16], [17], [18], [19], [20], [21]. We recently developed a hair cortisol assay to monitor long-term stress in captive chimpanzees [1]. Nevertheless, it is important that the procedures used to assess the welfare of animals be suitable for implementation in a variety of laboratory settings and that we are able to reliably measure hair cortisol levels over the long term without artifacts. Thus, it is necessary to identify potential confounding factors associated with the method used. Several technical factors may influence the analysis of steroid hormones. For example, the storage method and duration affect the hormone levels in feces and urine samples [22]. Hair cortisol can be stored on a long-term basis under ambient temperature conditions, as suggested by a study that compared cortisol levels in hair obtained from modern humans with that of mummies [23]. However, direct evidence concerning the effects of storage on hair cortisol stability is limited. Furthermore, the quantity and optimal number of samples per individual necessary to estimate basal hair cortisol levels must be considered. Although the collection of multiple samples increases reliability, it also increases the costs of sampling and analysis. Thus, it is necessary to achieve a balance between cost and reliability. The analysis of hair cortisol involves a number of steps prior to the immunoassay, such as collecting samples from a body region, grinding the samples, and extracting and drying the hormone. These factors have not been systematically assessed for their effect on hair cortisol levels. In our previous study, we found that body regions and hair color affected the hair cortisol results, as results obtained from the side of the body were higher than those obtained from other body parts, and hair cortisol levels obtained from white hair were higher than those obtained from black hair [1]. We tested several procedures for measuring cortisol levels in hair collected from captive chimpanzees to evaluate hormone stability over time and to identify the methodological factors that can affect the results of hair cortisol analysis, such as reproducibility, sampling, storage, grinding, extraction, and drying.

## Figures and Tables

**Table 1:** Hair cortisol concentrations obtained using different methods. Data of “Part-of-body” and “Hair color” were taken from Yamanashi et al. [1] for comparison.

Variable	Correlation	*M* (pg/mg hair)	SD	CV (%)
(a) Body part-arm only (*N* = 14)				
Arm1	0.70*	14.5	2.74	10.5
Arm2		14.6	4.03	
(a) Body part				
Arm	0.61–0.71*	16.8	4.90	17.9*
Side		20.8	7.44	
Back		19.4	6.11	
(b) Reproducibility (*N* = 22)				
First	0.92*	22.9	6.73	8.20
Second		23.9	7.93	
(b) Preserve 1 yr (*N* = 29)				
First	0.76*	15.9	3.47	7.40
Second		15.4	3.04	
(b) Preserve 2 yrs (*N* = 16)				
First	0.90*	14.5	2.83	5.10
Second		14.4	2.86	
(c) Fineness of homogenized hair (*N* = 19)				
Fine	0.85*	24.5	9.86	25.0*
Coarse		18.9	13.0	
(d) Hair weight (*N* = 6)				
20 mg	0.68–0.98	24.0	3.68	22.3*
10 mg		25.8	3.56	
5 mg		27.9	3.74	
2.5 mg		29.2*	3.81	
1 mg		40.8*	5.14	
(e) Extraction time (*N* = 11)				
1 h	0.73–0.82*	14.5	1.61	18.2*
8 h		18.0*	2.26	
24 h		20.6*	2.83	
(f) Drying (*N* = 26)				
Nitrogen gas	0.76*	15.3	3.09	6.80
Vacuum oven		14.8	2.65	
(–) Hair color	0.42			
Black		12.6	3.40	50.0*
White		26.5	6.91	

	Average (pg/mg hair)	SD	CV (%)
(g) Variances (*N* = 73)			
Within individuals	20.9	5.03	30.1
Between individuals			24.0

M, mean; SD, standard deviation; CV, coefficient of variation.

* *p* < 0.05.

## Appendix A Supplementary data

Correlation graphs of the method-validation tests. The solid line represents the fitting line, and the dashed lines indicate the 95% confidence interval.
