# Supplementary figures and images for: Analysis of hair cortisol levels in captive chimpanzees: Effect of various methods on cortisol stability and variability
Source: MethodsX. 2016 Jan 16;3:110–7. doi: 10.1016/j.mex.2016.01.004 (PMC4739149; doi:10.1016/j.mex.2016.01.004)

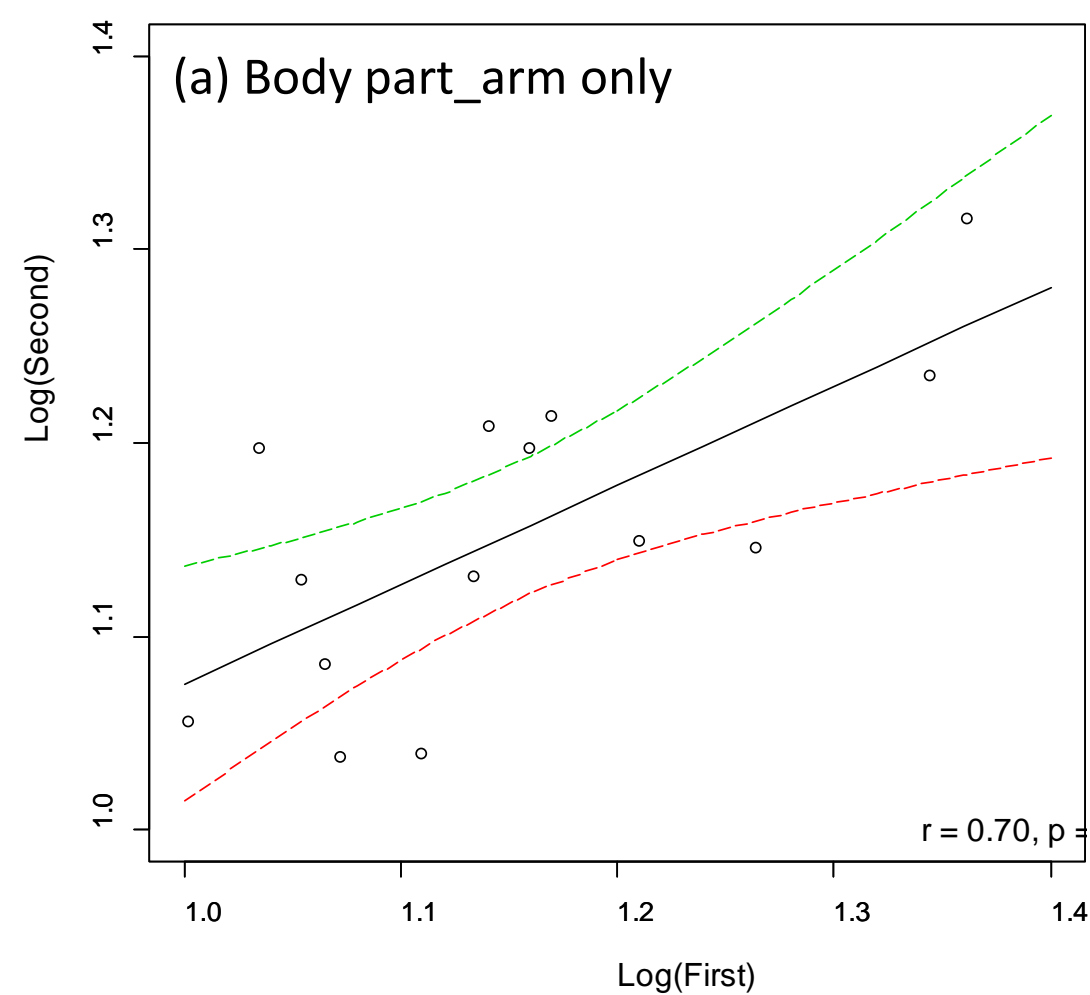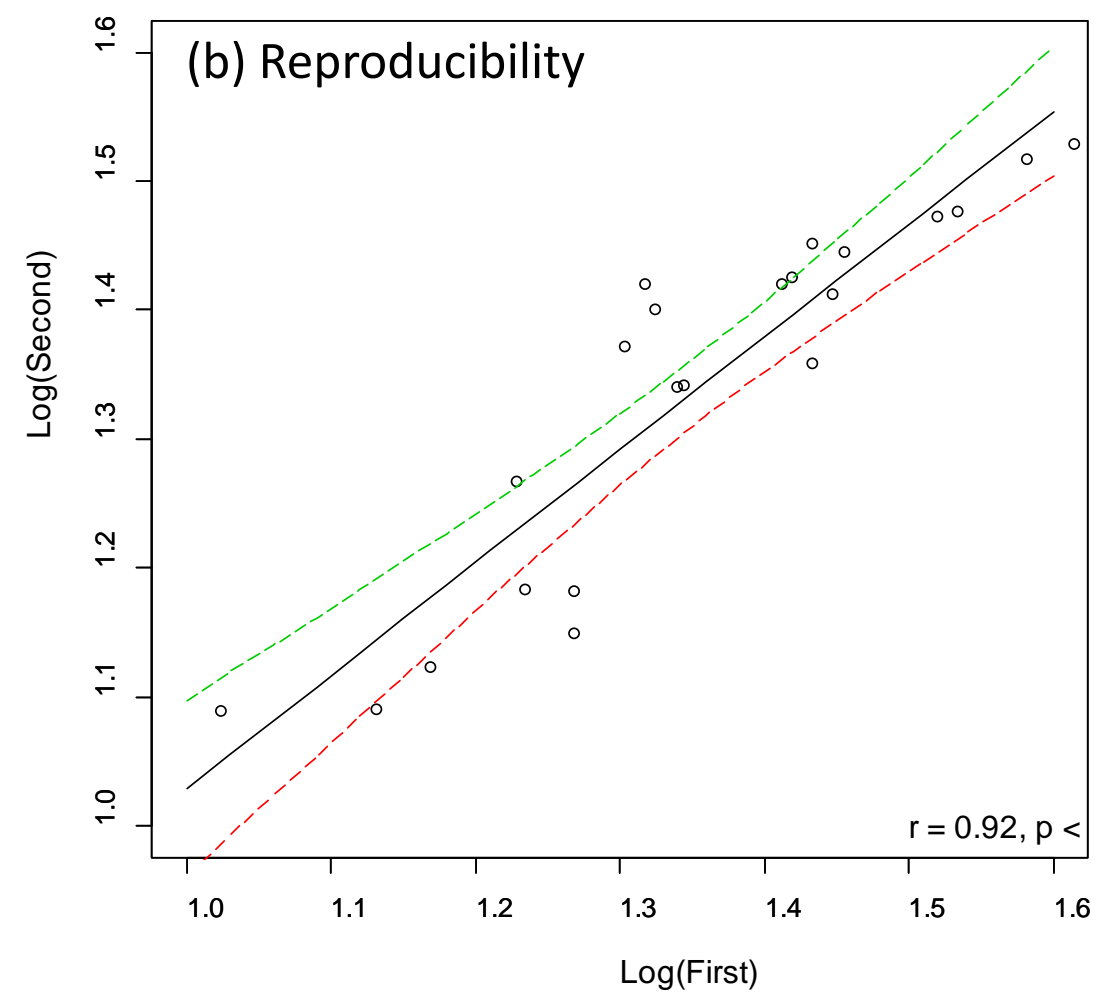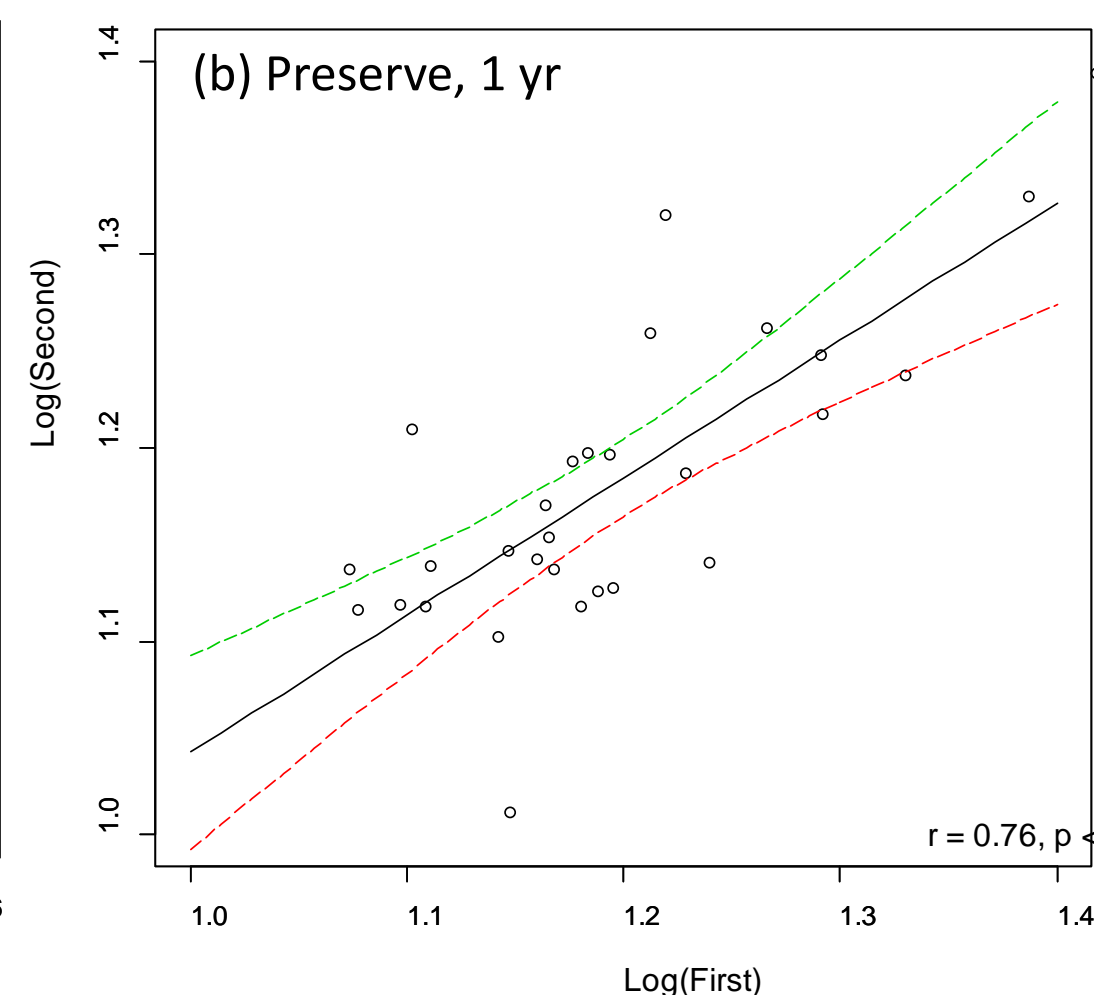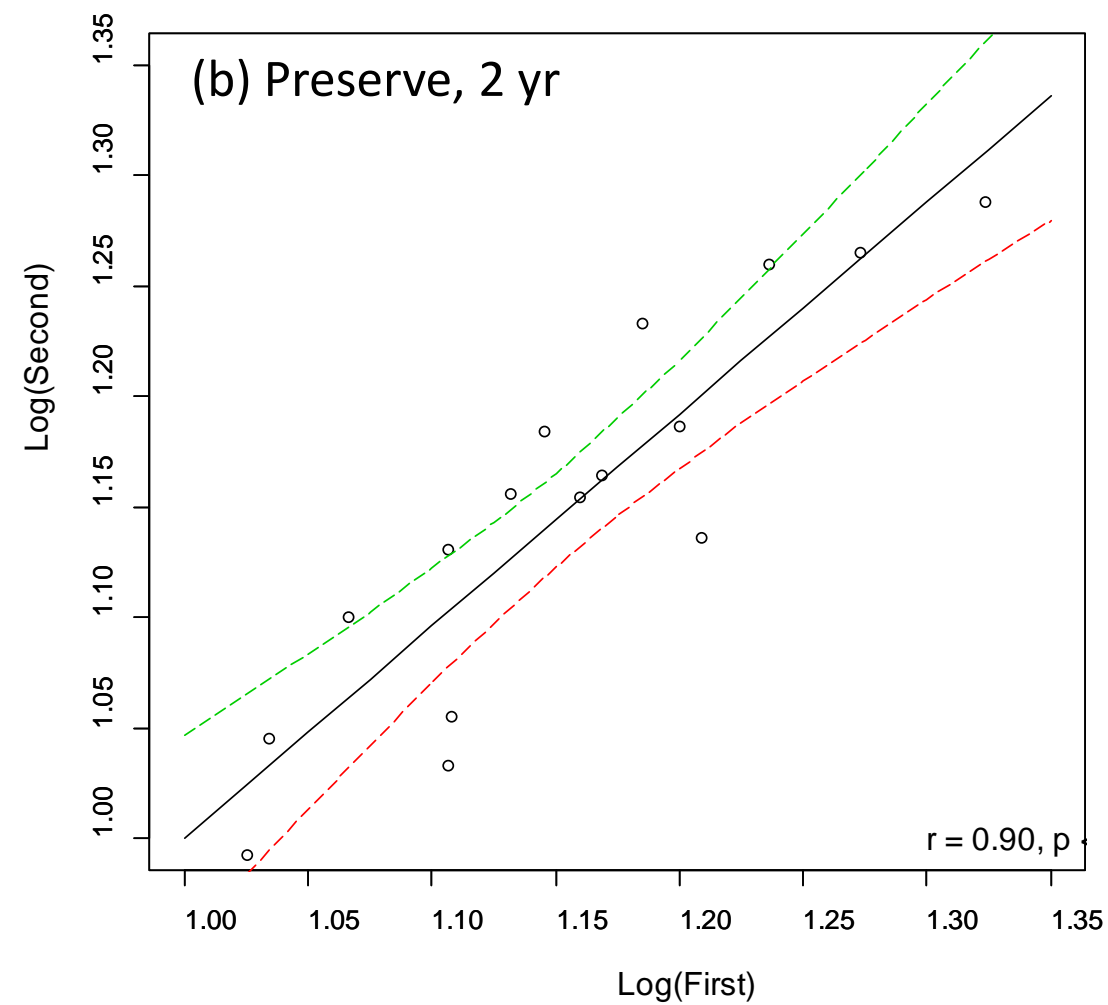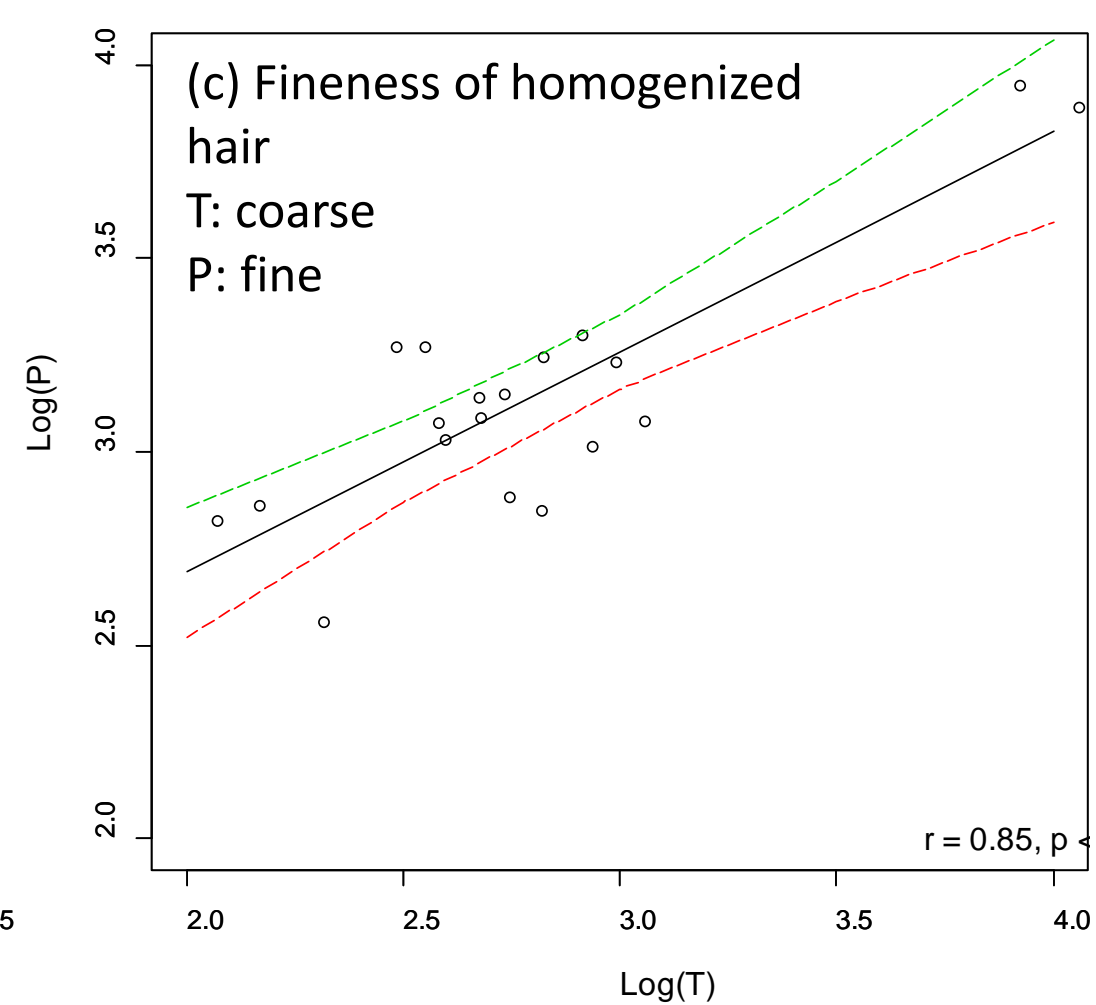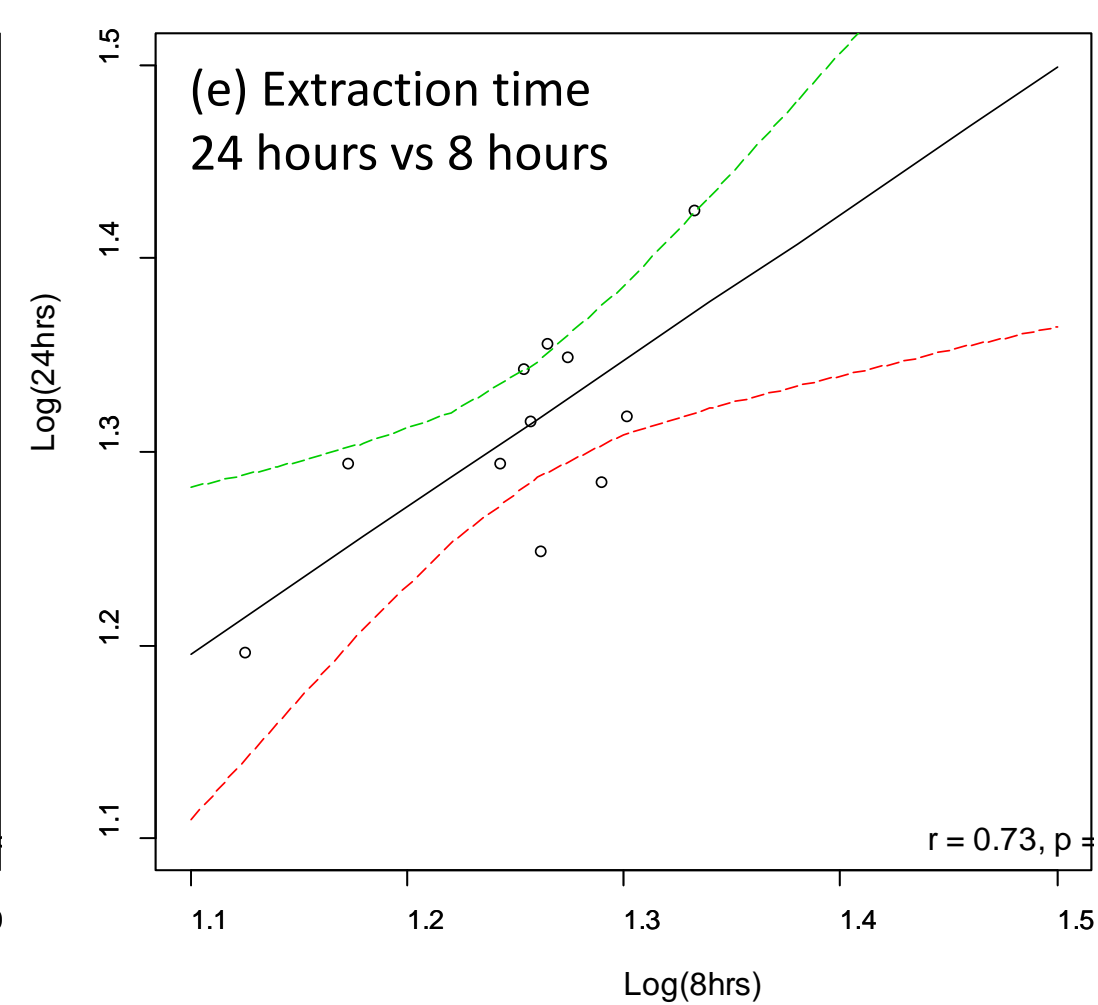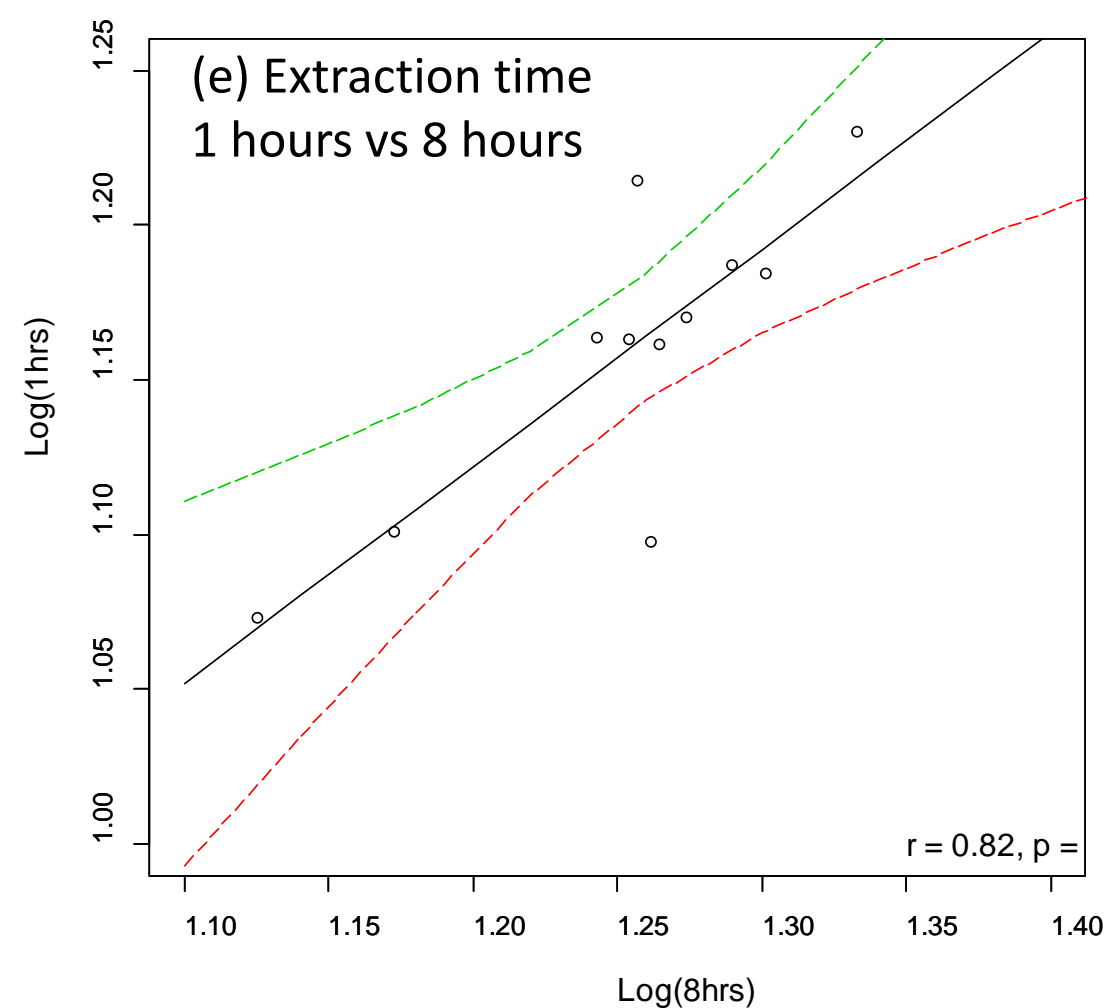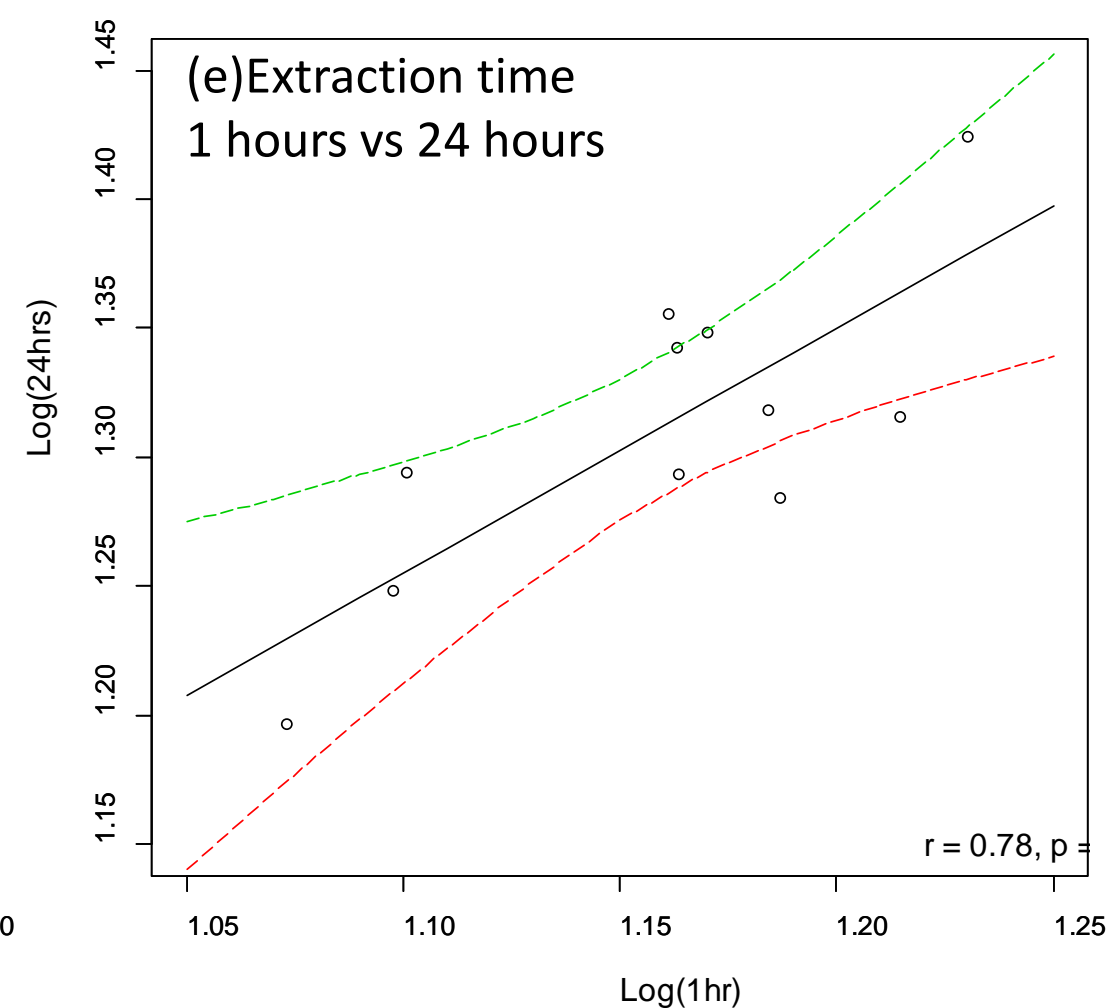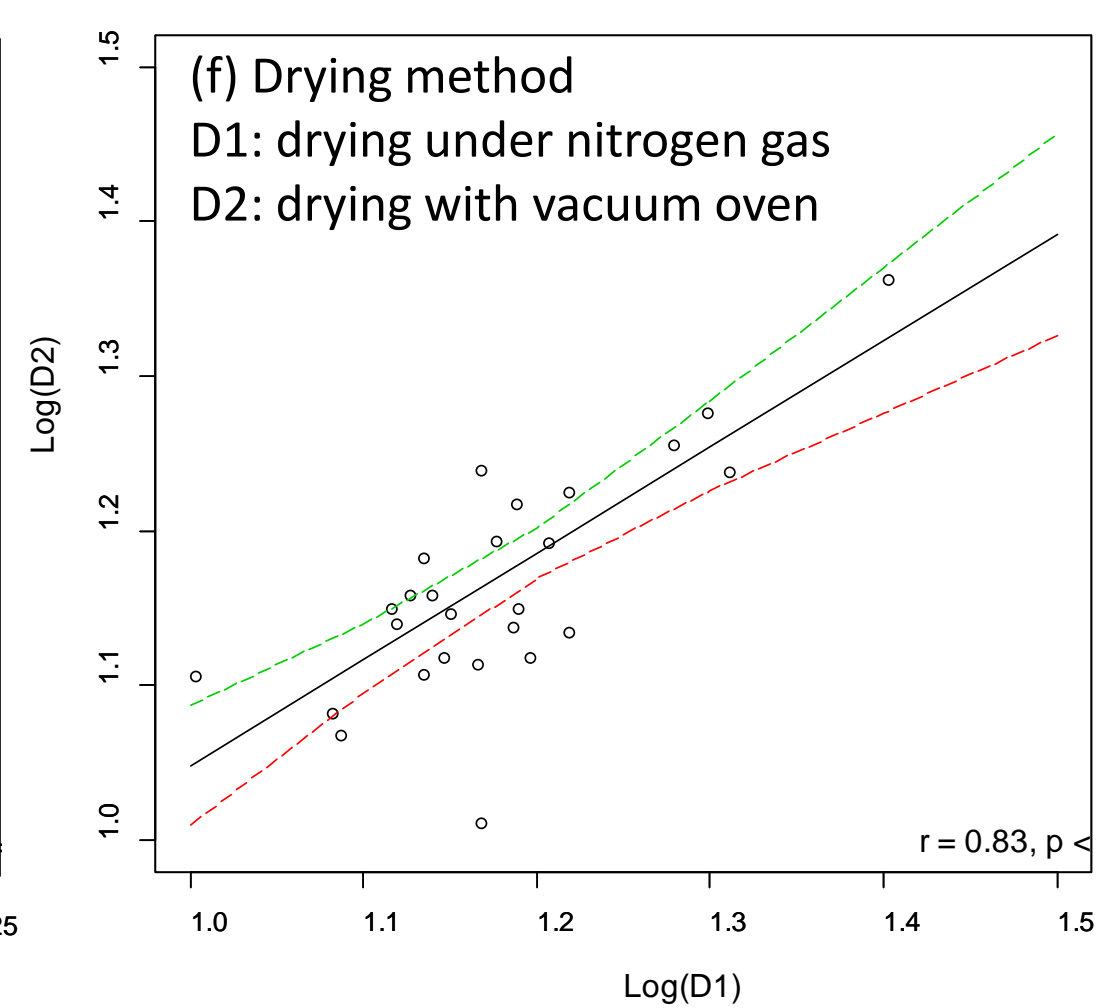

Supplement: Supplementary file 1 — Correlation graphs of the method-validation tests. The solid line represents the fitting line, and the dashed lines indicate the 95% confidence interval. [file mmc1.pdf]
